# Effect of Aggregate Type on Asphalt–Aggregate Adhesion and Its Quantitative Characterization

**DOI:** 10.3390/ma18153696

**Published:** 2025-08-06

**Authors:** Liuxiao Chen, Junlin Li, Hao Xiang, Jun Zhang, Enlin Feng, Lin Kong

**Affiliations:** 1 Shock and Vibration of Engineering Materials and Structures Key Laboratory of Sichuan Province, Southwest University of Science and Technology, Mianyang 621010, China; 2 College of Civil Engineering and Architecture, Southwest University of Science and Technology, Mianyang 621010, China; 3 National & Local Joint Engineering Research Center of Transportation and Civil Engineering Materials, Chongqing Jiaotong University, Chongqing 400074, China; 4 Sichuan Zhen Tong Inspection Co., Ltd., Mianyang 615499, China; 5 College of Civil Engineering, Southwest Jiaotong University, Chengdu 610031, China

**Keywords:** asphalt–aggregate adhesion, water boiling test, surface energy, image processing techniques

## Abstract

To study the effect of aggregate type on the adhesion between asphalt and aggregate, limestone, basalt, diabase, and 70# asphalt with SBS asphalt were selected. The mineral phase composition of the aggregates was analyzed by X-ray diffraction. The surface energy theory was used to calculate the adhesion work and the work of flaking. The modified water boiling method combined with image processing technology was used to quantitatively characterize the flaking behavior of the asphalt. The results show that the aggregate type is closely related to the asphalt–aggregate adhesion. The mineral compositions of different types of aggregates vary significantly, with limestone, being a strongly alkaline aggregate predominantly comprising CaCO_3_, exhibiting better adhesion with asphalt. The contact angle test and modified boiling method also yielded the same results, and the adhesion relationship with asphalt was limestone > basalt > diabase. Image processing technology effectively characterizes the spalling situation of asphalt and conducts quantitative analysis.

## 1. Introduction

Asphalt pavements have become the main pavement structures because of their advantages, such as low noise, traveling comfort, and easy construction and maintenance. As typical multiphase composite materials, the overall performance and durability of asphalt mixtures are highly dependent on the bond strength at the interface between the asphalt and the aggregate [1]. The interfacial bonding performance not only affects the mechanical response of the mixture but also directly determines the water damage resistance of the pavement and its long-term service life. Aggregates, as the main structural material in the mix (more than 90% by mass), have a significant influence on interfacial adhesion owing to their chemical composition, mineral properties, and surface morphology [2,3]. Therefore, it is crucial to study the effect of the aggregate type on asphalt–aggregate interfacial adhesion.

The formation of an interfacial system of asphalt mixtures is a dynamic bonding process of two-phase substances realized through physicochemical reactions. When asphalt interacts with mineral aggregates, its components migrate to the aggregate surface through mechanisms, such as penetration and diffusion, accompanied by surface energy interactions. The strength and properties of this interfacial bond are limited by the material composition and surface properties of the substrate on both sides [4,5]. Some of the mineral components of aggregates (SiO_2_, CaO, etc.) significantly modulate the migration rate of the colloidal and asphaltene components of asphalt at the microscopic level and determine the type of chemical bonding of the binding interaction and influence the adhesion to asphalt [6]. In engineering practice, the aggregate surface presents a multimineral composite structure. This heterogeneous interface produces a selective adsorption phenomenon when contacting asphalt, and its contact surface morphology is directly related to stress transfer efficiency and stripping resistance [7].

Traditional methods for evaluating the adhesion of asphalt–aggregate interfaces include boiling and water immersion. Relevant studies have shown [8,9] that the water boiling method is a simple and fast macro method to evaluate asphalt–aggregate adhesion; however, it can only be used as a rough indicator for qualitative evaluation because of its large subjective influence. In addition, the pull-off test is an intuitive method for evaluating the adhesion properties at the asphalt–aggregate interface. Cui [10] macroscopically evaluated the effects of multiple factors, such as aging asphalt content, rejuvenating agent, warm mixing agent, and various types of aggregates on asphalt–aggregate adhesion. Pang [11] investigated the effects of asphalt aging on the asphalt–aggregate interfacial adhesion by utilizing the pull-off test. At the microscale, Hou [12] investigated the adhesion between three recycled concrete aggregates and asphalt and the effect on the mix’s properties using surface free energy theory. Tu [13] quantitatively characterized the effect of water vapor on the bonding properties at the asphalt–aggregate interface by calculating the surface energy parameter. At the molecular/atomic scale, molecular dynamics (MD) simulations and atomic force microscopy (AFM) have been widely employed to analyze asphalt–aggregate bonding [14,15,16]. However, these microscopic-scale techniques cannot adequately capture the heterogeneous mineral distribution in macroscopic aggregates or the aging gradient in asphalt. In addition, Yin [17] explored the effect of the aggregate’s chemical composition on its adhesion. Wang [18] simulated the asphalt–aggregate adhesion model at different loading rates and found that the gradual delamination of the aggregate–asphalt interface at low speeds resulted in slight adhesion, whereas at high speeds, the large number of chemical bonds that break in a short period would improve the adhesion.

In the existing research, the influence mechanism of aggregates on the adhesion of asphalt–aggregate interfaces has not yet formed a systematic conclusion, and the traditional evaluation methods (such as the boiling method and pulling test) can reflect the macroscopic adhesion performance; however, they are limited by the subjectivity error and single-scale analysis, which makes it difficult to reveal the quantitative correlation between the mineralogical properties of aggregates and the interface interaction from a multidimensional perspective. In this study, a multiscale coupled quantitative characterization system is proposed to study the effect of aggregate type on asphalt–aggregate adhesion through X-ray diffraction (XRD) analysis, surface energy theory, a modified boiling method, and image processing techniques. The aim is to construct a cross-scale correlation framework of the mineralogical characteristics of aggregate–interfacial energy state–macroscopic peeling behavior to provide theoretical support for optimizing the design of asphalt mixtures and enhancing the durability of the interface.

## 2. Materials and Methods

### 2.1. Raw Materials

This study used the 70# asphalt (base asphalt, Penetration grade 60/80 dm) and Styrene–Butadiene–Styrene (SBS)-modified asphalt produced by Lu’ante Asphalt Technology Co., Chongqing, China. The aggregates used were for the local production of Mian yang City—limestone, basalt, and diabase—and particle sizes were in the range of 10–15 mm. The technical indices of asphalt and aggregate are listed in Table 1 and Table 2, respectively.

### 2.2. Experimental Methods

#### 2.2.1. X-Ray Diffraction

XRD is widely used as an analytical test method for determining the physical phase and crystallinity of a material, and can qualitatively analyze the mineral composition of aggregates. The test was conducted using an Empyrean diffractometer (PANalytical, Netherlands) with Cu Ka rays as the radiation source. The scanning parameters included a range of 5~90°, a speed of 8°/min, and a step size of 0.026°. The instrument operated at a voltage of 35 kv and a current of 35 mA, and the diffraction results were processed using the Jade 9 software to determine the mineral composition of the aggregates. Test each type of aggregate three times and take the average value.

#### 2.2.2. Contact Angle Test

The test was conducted using a Drop Shape Analysis 100 optical contact angle meter (KRUSS, Germany) to measure the static contact angle of formamide and distilled water on asphalt and aggregate by the seated drop method. The surface energy parameters of formamide and distilled water are shown in Table 3. Aggregate specimens were polished using raw stone cut to a size of 50 × 50 × 5 mm. Asphalt specimens were prepared by heating to a molten state, placing small amounts of drops on a slide, leveling them naturally in an oven at 160 °C for 2 min, and then cooling at room temperature for 2 h. Each sample underwent three independent contact angle determinations, where the arithmetic mean of triplicate measurements was designated as the representative contact angle value.

According to Good [19] and Fowkes [20], the surface tension of a solid/liquid consists of a dispersive component $\gamma^{d}$ and a polar component $\gamma^{p}$. Therefore, the surface free energy can be calculated using Equation (1).

(1) $$\gamma =\gamma^{p}+\gamma^{d}$$

For solid–liquid interfaces, the interfacial surface energy was calculated as

(2) $$\gamma_{sl}=\gamma_{s}+\gamma_{l}-2\sqrt{\gamma_{s}^{d}\gamma_{l}^{d}}-2\sqrt{\gamma_{s}^{p}\gamma_{l}^{p}}$$

This is obtained by combining the Young-type equations

(3) $$\gamma_{l}(1+\cos\theta )=2\sqrt{\gamma_{s}^{d}\gamma_{l}^{d}}+2\sqrt{\gamma_{s}^{p}\gamma_{l}^{p}}$$

Here, $\gamma_{sl}$ represents the surface free energy of the solid–liquid interface; $\gamma_{s}$ and $\gamma_{l}$ represent the surface free energies of the solid/liquid, respectively; $\gamma_{s}^{d}$, $\gamma_{s}^{p}$, $\gamma_{l}^{d}$, and $\gamma_{d}^{p}$ represent the dispersive and polar components of the surface free energies of the solid/liquid, respectively; and θ denotes the contact angle.

Surface free energy theory is used to evaluate the adhesion between solids and liquids with and without water by exfoliation and adhesion work, respectively. The asphalt–aggregate inter-adhesion work ($W_{ae}$) and flaking work ($W_{awe}$) were calculated using Equations (4) and (5).

(4) $$W_{ae}=2\sqrt{\gamma_{a}^{d}\gamma_{e}^{d}}+2\sqrt{\gamma_{a}^{p}\gamma_{e}^{p}}$$

(5) $$W_{awe}=2(\sqrt{\gamma_{a}^{d}\gamma_{w}^{d}}+\sqrt{\gamma_{a}^{p}\gamma_{w}^{p}}+\sqrt{\gamma_{e}^{d}\gamma_{w}^{d}}+\sqrt{\gamma_{e}^{p}\gamma_{w}^{p}}-\sqrt{\gamma_{a}^{d}\gamma_{e}^{d}}-\sqrt{\gamma_{a}^{p}\gamma_{e}^{p}})$$

Here, $\gamma_{a}^{d}$, $\gamma_{e}^{d}$, $\gamma_{w}^{d}$, $\gamma_{a}^{p}$, $\gamma_{e}^{p}$, and $\gamma_{w}^{p}$ represent the dispersion and polarity components of asphalt, aggregate, and water, respectively.

#### 2.2.3. Improved Boiled Water Test

The boiling water test for evaluating the asphalt stripping area is subject to subjective judgment, making accurate quantitative analysis difficult. To address this limitation, this study extended the boiling duration and employed a precision balance (0.1 mg resolution) to measure the specimen mass at 5 min intervals during boiling. The mass loss rate of the asphalt film was calculated until the variation in mass loss per 5 min interval fell below 0.5%. The mass of the clean aggregate was recorded as $M_{0}$; the mass of the wrapped asphalt membrane was recorded as $M_{1}$. After boiling, the sample was air-dried on a rack for 15 min and then weighed to obtain its mass, denoted as $M_{2}$. The rate of loss of asphalt mass $W_{h}$ was calculated using Equation (6). Four particles are selected for each aggregate type, with the average value designated as $W_{h}$.

(6) $$W_{h}=\frac{M_{1}-M_{2}}{M_{1}-M_{0}}\times 100\%$$

The positive and negative spalling surfaces of the aggregates after boiling were obtained by a high-definition camera and imported into Image-Pro Plus (IPP) software for background rejection and stain recognition processing to identify spalling and non-spalling areas. The asphalt film spalling rate was calculated using Equation (7) to quantitatively evaluate the asphalt–aggregate adhesion.

(7) $$P_{r}=\frac{S_{1}}{S_{1}+S_{2}}\times 100\%$$

Here, $P_{r}$ denotes the asphalt film spalling rate, and $S_{1}$ and $S_{2}$ denote the spalled and unspalled areas, respectively.

## 3. Results and Discussion

### 3.1. XRD Results Analysis

**Figure 1:**
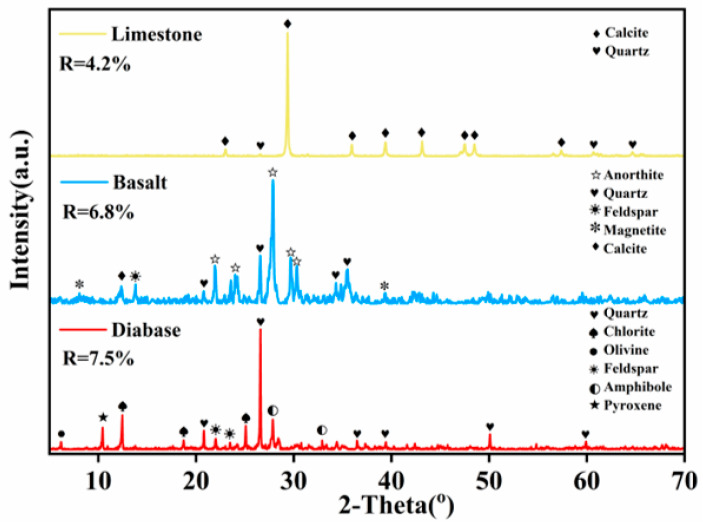
XRD diffractogram.

Figure 1 shows the XRD test results of the aggregate, in which the limestone material phase shows significant characteristic peaks at 2θ of 29° and no obvious heterogeneous peaks, which mainly consist of calcite (CaCO_3_) with a small amount of quartz. The low R-wp value of limestone reflects its precise fitting dominated by a single phase, and a few quartz peaks can also be clearly analyzed. Compared with limestone, basalt and diabase have more characteristic peaks and more miscellaneous peaks in their material phases, and their mineral compositions are more complex, presenting higher R-wp values. This might be related to the formation processes of the two substances, both of which are basic igneous rocks formed by the cooling of magma. The formation environments are different: diabase is a shallow intrusive rock, while basalt is an ejecta rock, which affects the crystallization process and the differentiation of minerals. However, the diversity of mineral compositions of both materials originates from the combined effects of magma evolution, crystallization process, and geological environment.

**Figure 2:**
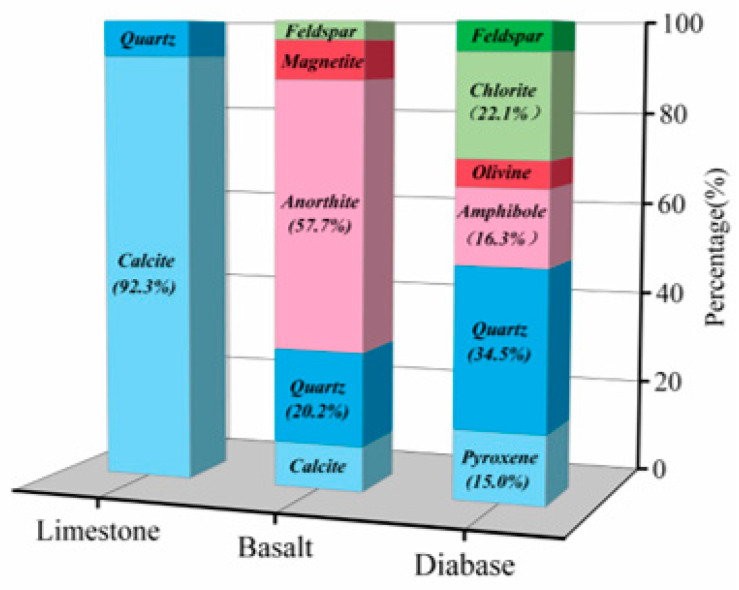
Aggregate mineral composition.

Figure 2 shows the mineral composition of the aggregate, which was calculated from the peak area of the characteristic peaks of the XRD curve. The main mineral composition of limestone is calcite, accounting for 92.3%. The mineral composition of basalt mainly consists of calcium feldspar and quartz, accounting for 57.7% and 20.2%, respectively, while the mineral composition of diabase contains a large amount of quartz, chlorite, hornblende, and pyroxene, which account for 34.5%, 22.1%, 16.3%, and 15%, respectively. Due to the poor adhesion between quartz and asphalt, the quartz phase is prone to causing water damage in asphalt mixtures. Although sodium feldspar can form a strong bond with asphalt under anhydrous conditions, it is prone to failure in the presence of water. Related studies have shown that feldspar may result in bond failure at the asphalt–aggregate interface [21,22]

### 3.2. Contact Angle Test Results

#### 3.2.1. Analysis of Contact Angle

**Figure 3:**
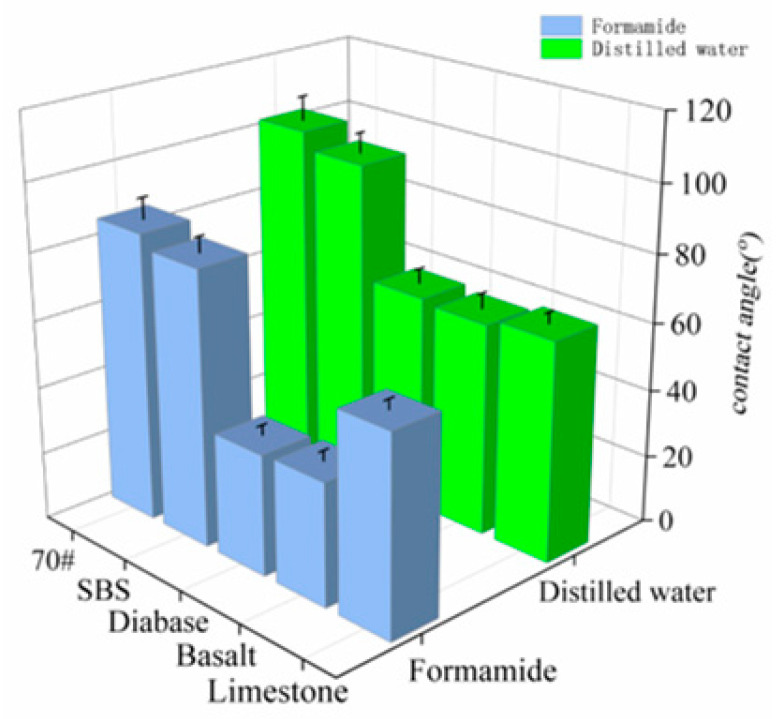
Contact angle test results.

**Figure 4:**
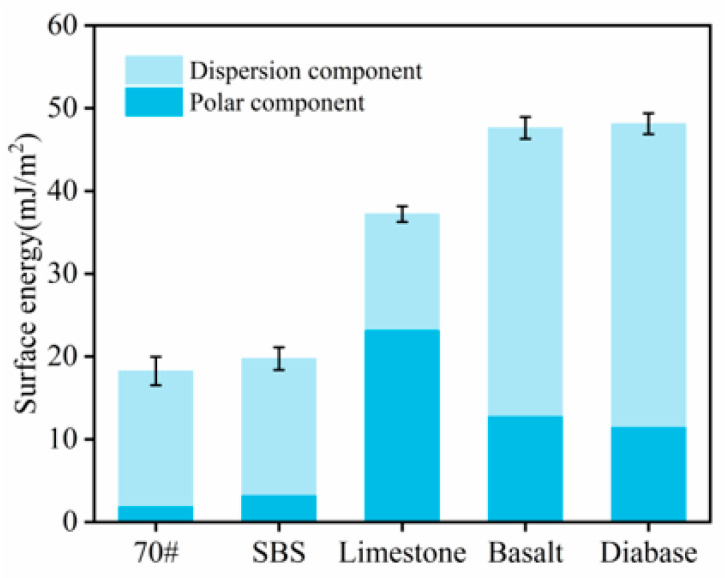
Surface energy parameters.

Figure 3 shows the results of the contact angle test of the asphalt and aggregate with the measured liquid, and the surface energy parameters of each asphalt and aggregate were calculated by substituting the obtained results into Equation (3), as shown in Figure 4.

Figure 3 shows the results of determining the contact angle of the liquid with asphalt and aggregate. The contact angles of water with asphalt were all greater than 90°, whereas the contact angles with the aggregate were all less than 70°. This is because water is a strong polar material, and the polar component of the surface energy component accounts for the main part of the surface energy component; as shown in Figure 4, the asphalt surface energy in the polar component accounts for a small proportion of the non-polar material, and polarity differences result in difficulty in terms of infiltrating the surface of the asphalt, resulting in a larger contact angle.

Three types of aggregate surface energies of the polar component of the surface energy accounted for a larger proportion than the asphalt. The aggregates had better adhesion to water, and the contact angle with the water was also smaller. Limestone contains 92.3% calcite, and CaCO_3_ is an alkaline mineral with a significant polar component; therefore, the polar component is larger than that of basalt and diabase. Relative to the base asphalt, the SBS-modified asphalt polarity component increased, mainly because the addition of modifiers made the base asphalt, in some of the nonpolar saturated points and aromatic points, exhibit a strong polarity for the gum and asphaltene. Water and aggregate polarity differences are smaller than asphalt, resulting in water molecules preferentially adsorbed on the aggregate surface, through the “replacement effect” to destroy the asphalt–aggregate interface; the greater the difference in polarity, the stronger the replacement.

#### 3.2.2. Adhesion and Peeling

**Figure 5:**
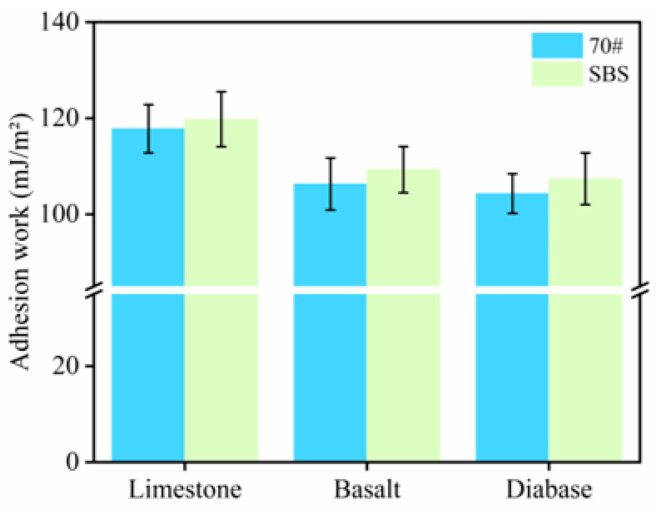
Adhesion work between asphalt and aggregate.

**Figure 6:**
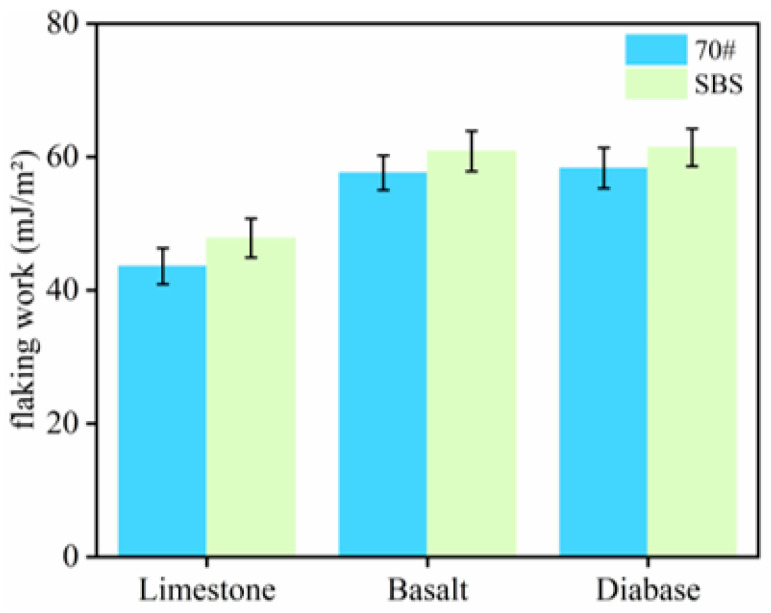
Flaking work between asphalt and aggregate.

The work of adhesion between the asphalt and aggregate and the work of spalling were calculated according to Equations (4) and (5), and the results are shown in Figure 5 and Figure 6.

As shown in Figure 5, the magnitude of the adhesion work between the three types of aggregates, the 70# base asphalt, and SBS-modified asphalt is limestone > basalt > diabase. Higher adhesion work implies stronger asphalt–aggregate bonding. This indicates that, under anhydrous conditions, among the three types of aggregates, limestone exhibited the best adhesion with asphalt. Under the same type of aggregate, the adhesion between the SBS-modified asphalt and the aggregate was better than that of the 70# asphalt, which was related to the excellent adhesion of the SBS-modified asphalt. Under water conditions, the magnitude of the flaking work between the asphalt and aggregate was basalt > diabase > limestone, as shown in Figure 6. The larger the absolute value of the spalling work, the greater the possibility of stripping between asphalt and aggregate; thus, the bond between limestone and asphalt is stronger.

Based on the results of the adhesion and spalling work among asphalt aggregates, limestone exhibits the strongest adhesion with asphalt, followed by basalt, while diabase shows the weakest adhesion. This is because limestone mainly comprises the alkaline mineral CaCO_3_, which has a high surface hydroxyl density and strong polarity and can form hydrogen and chemical bonds with polar molecules (e.g., colloid and asphaltene) in asphalt, thus enhancing the adhesion work [23]; whereas, both basalt and diabase contain a large amount of quartz, and the hydroxyl density of the SiO_2_ surface is low and acidic, with a weaker polarity component. In addition, the hydrophobic quartz surface is prone to competition with water molecules for adsorption, which accelerates interfacial exfoliation [24]. Thus, the aggregate type has a large influence on the adhesion between asphalt and aggregate.

### 3.3. Results of the Modified Boiling Method

**Figure 7:**
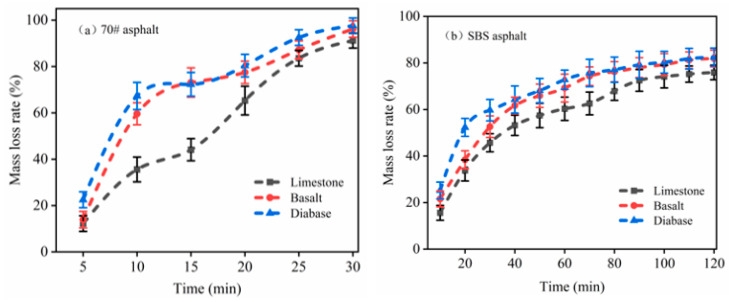
Asphalt mass loss rate: (**a**) 70# asphalt; (**b**) SBS asphalt.

The spalling rate was characterized by weighing the rate of asphalt mass loss of the test samples every 5 min by adjusting the method based on water boiling. The asphalt mass loss rate for each sample is shown in Figure 7.

Figure 7 shows the mass loss rate of the 70# asphalt, SBS asphalt, and SBS-modified asphalt owing to their excellent cohesion and better adhesion with the aggregate, resulting in a lower rate of asphalt mass loss than the matrix asphalt. Regardless of whether the 70# matrix asphalt or SBS-modified asphalt was used, the asphalt mass loss rate of the samples showed a trend of first increasing and then decreasing. Taking 70# asphalt as an example, during the initial phase of the boiling water test, the cohesive work of the asphalt itself is lower than the adhesive work between the asphalt and aggregate. Under the dynamic water pressure generated by boiling, asphalt stripping primarily involves the detachment of excess asphalt from the aggregate surface, resulting in a reduction in asphalt film thickness and a relatively high loss rate; that is, the asphalt film thickness decreases, and the rate of loss increases. With increasing soaking time, the thickness of the asphalt film decreased, and the rate of flaking gradually decreased. The initial water content between the asphalt aggregates is almost zero because of the different internal and external water gradients, and under the action of dynamic water flushing, the water penetrates the interface along the pores. The polarity of water compared to asphalt is stronger, and thus the water will gradually replace the asphalt on the aggregate surface. The more water infiltration, the stronger the replacement effect, until the interface at the water reaches equilibrium, and the asphalt spalling on the aggregate surface gradually tends to stabilize. Wang’s [25] findings suggest that under humid conditions, water molecule infiltration at the asphalt–aggregate interface leads to significant degradation of adhesive forces, consequently impairing interfacial bonding performance.

From the asphalt spalling results, the pattern of the asphalt mass loss rate for the different aggregate types is diabase > basalt > limestone. The limestone aggregate shows less asphalt spalling because its main mineral component is CaCO_3_, which has a positive effect on water sensitivity. The limestone aggregate material is mainly alkaline, whereas the asphalt is acidic; therefore, it has better adhesion with asphalt. As indicated in the previous section, the diabase mineral composition contains a large amount of quartz. Its main component is SiO_2_, and silica results in asphalt and aggregate bonding with a reduction in adhesion, which is not conducive to the adhesion between the aggregate and asphalt; thus, diabase and asphalt have the worst adhesion effect. Su [1] reported that limestone and basalt exhibit superior asphalt adhesion due to their higher content of alkaline oxides, whereas andesite demonstrates poorer adhesion with asphalt owing to its lower alkaline oxide content and higher SiO_2_ concentration. Notably, the work of adhesion between alkaline oxides and asphalt was found to be significantly greater than that between acidic oxides and asphalt.

**Figure 8:**
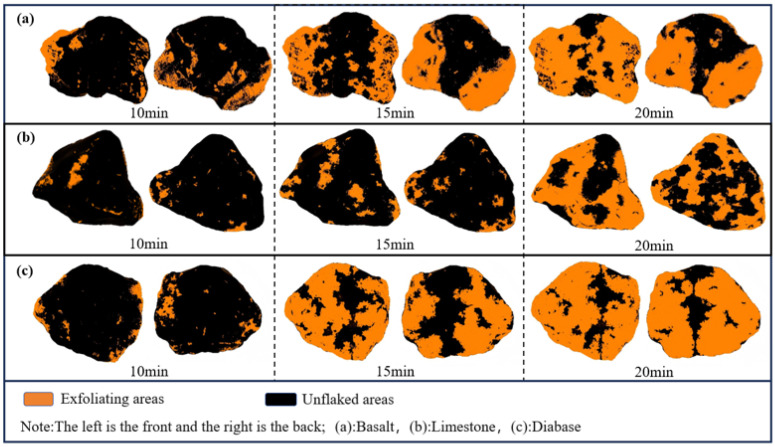
Schematic of image processing (70# asphalt).

Using IPP software, we performed threshold segmentation on the modified boiling water test results through an automatic Otsu algorithm (preliminary screening of 8-bit grayscale images) followed by manual HSV refinement, with a minimum analysis area of 5 mm^2^. The staining areas on both sides of the sample were identified, and the inclusion criteria were pixel intensity <100 and compliance with the HSV range. Then, the pixel area of the stained area is extracted. The image uses a 10 mm scale, and 1 mm = 48 pixels. The spalling process of the 70# asphalt test sample after image processing is shown in Figure 8. Based on the experimental observations and images, the asphalt film is most likely to peel off at the corners of the aggregates. As the boiling time increases, water will gradually replace the residual asphalt in the peeled areas along the already peeled areas.

**Figure 9:**
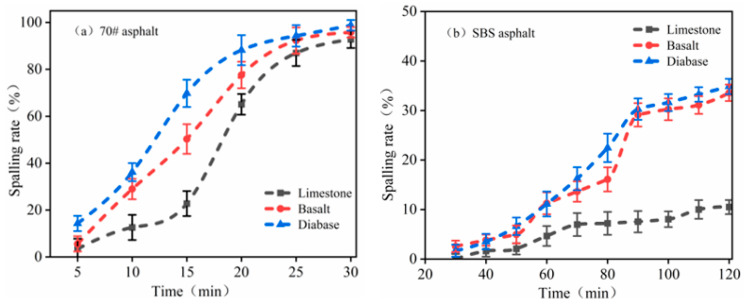
Asphalt spalling rate: (**a**) 70# asphalt; (**b**) SBS asphalt.

Figure 9 shows the asphalt spalling rate after image processing. The flaking rate curve shows a monotonically increasing trend, in line with the asphalt–aggregate interface water damage dynamic development law. The 70# asphalt flaking rate exceeded 90%, and only a small amount of asphalt residue was present on the surface of the aggregate. However, the SBS-modified asphalt demonstrated minimal stripping during the initial 55 min immersion, with rates consistently below 10%. Even with prolonged testing, the stripping rates remained relatively low, ultimately ranging between 10.5% and 34.8%. These findings clearly indicate that the boiling water test is unsuitable for evaluating the bond characteristics between SBS-modified asphalt and aggregates.

Based on the asphalt spalling-rate results, limestone and asphalt had better adhesion properties, followed by basalt aggregate and diabase, which is consistent with the previous asphalt mass loss rate results of the law. However, the flaking rate of the SBS-modified asphalt is far from the previous asphalt mass loss rate, which may be caused by the thick asphalt film wrapped around the aggregate surface when fabricating the specimens using the boiling method. In contrast, the results processed using the image were more representative of a real asphalt film flaking situation.

## 4. Conclusions

In this study, the effect of aggregate type on asphalt–aggregate adhesion is systematically revealed from the microscopic to the macroscopic scale by adopting a multiscale coupled quantitative characterization system through XRD analysis, surface energy theory, a modified water boiling method, and image processing techniques.

(1) The mineral compositions of the three aggregates were tested separately by XRD. Limestone has a single-mineral composition, mainly calcite and a small amount of quartz; basalt and diabase have complex mineral compositions, and the quartz and feldspar compositions account for a relatively large proportion, which is not conducive to adhesion between the asphalt and aggregates.

(2) Adhesion and flaking work, calculated based on contact angle tests, showed that SBS asphalt and 70# asphalt exhibited the strongest adhesion with limestone under water-free conditions. In the presence of water, their flaking work was the lowest, further confirming that limestone has the strongest ability to resist water damage. In addition, SBS-modified asphalt outperformed 70# bitumen due to its elevated polarity fraction, resulting in better adhesion.

(3) By modifying the water boiling method and measuring the rate of asphalt mass loss, combined with an image processing technique to quantify the spalling area, it was found that limestone asphalt exhibited the lowest rate of spalling, while diabase showed the highest. This method effectively characterized the macroexfoliation behavior of asphalt on aggregate surfaces, reduced subjective errors, and verified the influence of the mineral composition and surface energy theory on adhesion performance.

This study utilized three commonly used aggregate types and two asphalt binders to evaluate asphalt-aggregate bonding performance. However, the selected materials do not fully represent the adhesion behavior of all alkaline, neutral, and acidic aggregates with asphalt. Moreover, the effect of asphalt aging on interfacial bonding properties was not considered. Further research should investigate how aggregate alkalinity/acidity and asphalt aging degree influence asphalt–aggregate adhesion.

## Figures and Tables

**Table 1:** Technical specifications of asphalt.

Type	Penetration (0.1 mm)	15 °C Extension (cm)	Softening Point (℃)
70# Asphalt	68.0	114.2	51.5
SBS asphalt	52.8	36.8	77.0

**Table 2:** Aggregate technical specifications.

Type	Water Absorption (%)	Bulk Relative Density	Apparent Density (g·cm^−3^)	Crushing Value (%)	Abrasion Value (%)
Limestone	0.54	2.701	2.749	16.9	17.9
Basalt	0.56	2.892	2.978	9.6	6.4
Diabase	0.34	2.674	2.642	10.9	18.6

**Table 3:** Determination of liquid surface energy parameters.

Liquid Type	Surface Energy Parameters		
	γ (mJ/m^2^)	$\gamma^{p}$ (mJ/m^2^)	$\gamma^{d}$ (mJ/m^2^)
Carboxamide	58.2	18.7	39.5
Distilled Water	72.8	51.0	21.8

## Data Availability

The original contributions presented in this study are included in the article. Further inquiries can be directed to the corresponding author.
